# Supplementary material for: Content-rich biological network constructed by mining PubMed abstracts
Source: BMC Bioinformatics. 2004 Oct 8;5:147. doi: 10.1186/1471-2105-5-147 (PMC528731; doi:10.1186/1471-2105-5-147)
Supplement: Additional File 5 — The original Chilibot query results of the term "long-term potentiation (LTP)" and 22 other terms, limiting the latest references analyzed to the years 1990, 1995, 2000, and 2004. [file 1471-2105-5-147-S5.bz2 › chilibotAdditionalFile5/ltp2004/html/CAMKII_SYNAPTOTAGMIN.html]

 


 **CAMKII** and **SYNAPTOTAGMIN** 
  
Found 5 abstracts in PubMed,  **5 abstracts were retrieved and analyzed**.  


---

 Search Google  |
 PDF files only 
|  EDU domain only 

---

**Interactive relationship** (e.g. stimulation, inhibition, etc)

- All 5 HT reuptake blockers that we used, which induce an increase in  **CaMKII**  activity and autophosphorylation, also caused a large 2 3 fold increase in the calcium calmodulin dependent post hoc phosphorylation of  **synaptotagmin** .  Ref: 9016342 Mol Pharmacol, 1997
- SyntagI, the purified, recombinant, cytoplasmic domain of rat  **synaptotagmin**  I, was an effective substrate in vitro for calcium calmodulin dependent protein kinase II  **CaMKII** , protein kinase C PKC, and casein kinase II caskII.  Ref: 10461881 J Neurochem, 1999

**Parallel relationship** (e.g. studied together, co-existance, homology, etc.)

- To investigate the changes induced in presynaptic protein phosphorylation by 5 HT reuptake blockade and concomitant  **CaMKII**  up regulation, we analyzed two major  **CaMKII**  presynaptic substrates synapsin I and  **synaptotagmin** .  Ref: 9016342 Mol Pharmacol, 1997
- **CaMKII**  syntaxin complexes were also detected in synaptosomes by immunoprecipitation, and when reconstituted in vitro, they recruited larger amounts of  **synaptotagmin**  and SNAP 25 than syntaxin alone.  Ref: 11978810 J Neurosci, 2002
- This finding establishes a link between the facilitation of transmitter release induced by antidepressant drugs and the phosphorylation of  **synaptotagmin**  by  **CaMKII** .  Ref: 9016342 Mol Pharmacol, 1997
- Thus, drugs known to induce an increase in 5 HT release simultaneously induce an increase in the activity of presynaptic  **CaMKII**  and in the phosphate incorporation post hoc by a major  **CaMKII**  substrate in synaptic vesicles  **synaptotagmin** .  Ref: 9016342 Mol Pharmacol, 1997
- The results suggest the possibility that the phosphorylation of  **synaptotagmin**  I by  **CaMKII**  and PKC contributes to the mechanism s by which these two kinases regulate neurotransmitter release.  Ref: 10461881 J Neurochem, 1999
